# P02.136. A randomized controlled trial for the use of qigong in the treatment of pre and mild essential hypertension

**DOI:** 10.1186/1472-6882-12-S1-P192

**Published:** 2012-06-12

**Authors:** J Park, S Hong, T Park, Y Liu, J Kim, T Kim, A Kim, S Jung, H Park, S Choi

**Affiliations:** 1 Korea Institute of Oriental Medicine, Daejeon, Republic of Korea; 2 College of Oriental Medicine, Dongeui University, Pusan, Republic of Korea; 3 Qigong Treatment Center, Dongeui Medical Center, Pusan, Republic of Korea

## Purpose

Hypertension is a risk factor for cardiovascular disease, and the prevalence of hypertension tends to increase with age. Current treatments for hypertension have side effects and poor adherence. Qigong has been studied as an alternative therapy for hypertension; however, the types of qigong used in those studies were diverse, and there have not been many well-designed randomized controlled trials. Our objective is to evaluate the effects of qigong on blood pressure, health status and hormone levels for pre- or mild hypertension.

## Methods

Forty subjects with pre- or mild hypertension were randomized to either the qigong exercise group or the non-treated group. Participants in the qigong group conducted qigong exercises 5 times per week for 8 weeks, and participants in the non-treated group maintained their current lifestyle, including diet and exercise. The use of antihypertensive medication was not permitted. The primary endpoint was a change in patient blood pressure. Secondary endpoints were patient health status (as measured by the MYMOP2 questionnaires) and changes in hormone levels.

## Results

Of the 40 participants that were randomized, 35 completed the study. Systolic and diastolic blood pressures were significantly decreased after qigong treatment compare to baseline only in the qigong group (p<.001 in SBP, p<.0001 in DBP). In the non-treated group, there was no significant difference in blood pressure. Change of blood pressure between the qigong and the non-treated group was significant (p<.01 in SBP and DBP). The score of MYMOP2 showed a more significant decrease in the qigong group than the non-treated group (p=.035). Any differences in the hormones renin, angiotensin, cortisol, or norepinephrine were not significant between the two groups.

## Conclusion

Qigong appears safe and has a positive effect on blood pressure and health status in pre and mild hypertension patients. Further long-term studies with a larger number of subjects are warranted.

